# Zero Bias Operation: Photodetection Behaviors Obtained by Emerging Materials and Device Structures

**DOI:** 10.3390/mi13122089

**Published:** 2022-11-27

**Authors:** Juhyung Seo, Yeong Jae Kim, Hocheon Yoo

**Affiliations:** 1 Department of Electronic Engineering, Gachon University, Seongnam 13120, Republic of Korea; 2 Korea Institute of Ceramic Engineering and Technology, Ceramic Total Solution Center, Icheon 17303, Republic of Korea

**Keywords:** photodetectors, thin-films, photovoltaics, phototransistors, photodiodes

## Abstract

Zero-biased photodetectors have desirable characteristics for potentially next-generation devices, including high efficiency, rapid response, and low power operation. In particular, the detector efficiency can be improved simply by changing the electrode contact geometry or morphological structure of materials, which give unique properties such as energy band bending, photo absorbance and electric field distribution. In addition, several combinations of materials enable or disable the operation of selective wavelengths of light detection. Herein, such recent progresses in photodetector operating at zero-bias voltage are reviewed. Considering the advantages and promises of these low-power photodetectors, this review introduces various zero-bias implementations and reviews the key points.

## 1. Introduction

Photodetectors detect broadband light or other selective wavelengths. As the most frequently used photodetectors, semiconductor-based devices convert light to electrical signals based on the photoelectric effect [1,2,3]. For example, a photodiode is based on a p–n junction structure that consumes light energy to produce an electric current [4,5,6]. The photoreactivity can be enhanced by designing the photodiode to operate under a reverse bias region, where depletion occurs at the interface between the p- and n-sides. Photo-excited electron–hole pairs can be formed based on the bias condition of the external reverse voltage. These carriers are separated by energy band bending through the reverse voltage bias, contributing to the drift current flowing.

Another example is asymmetric semiconductor morphological properties or electrode variations [7,8,9,10,11]. The asymmetric electrode contacts or the use of an asymmetric electrode material produces an internal potential difference due to the difference in the charge injection area or work function between the source and the drain or the anode and the cathode. This potential difference can induce the flow of generated charge carriers.

Significant efforts on the development of the mentioned photodiodes have been made based on various materials, such as silicone [12,13,14], organic semiconductors [15,16,17], two-dimensional materials [18,19,20,21,22,23,24,25,26], metal oxides [27,28,29], and perovskites [30,31,32,33]. In addition, quantum dots [34,35], quantum wells [36], and single-element 2D materials [37] have also been extensively researched to integrate into zero-bias photodetectors.

Meanwhile, the phototransistor is based on a three-terminal structure [38,39,40]. The device can detect light simultaneously with the gate electrode and amplify the sensed light signal because an additional terminal provides a gating field effect in the channel between the contact electrodes [41]. With this amplification of the light-sensing signal, the phototransistors have attracted considerable attention, and the implementation of phototransistors has been attempted with various materials: organic semiconductors [42,43], transition-metal dichalcogenides [44,45], metal oxides [46,47], carbon nanotubes [48,49], and graphene [50,51].

On the other hand, low-power operation of photoreaction is required. The development of ultra-low-power operation is important because the number of sensing applications required increases significantly. Even these applications require real-time monitoring, which consumes continuous electricity. Along this line, attempts on zero-bias operation photodetectors have been made [52,53,54,55]. Rather than applying voltage bias to photodetectors, these attempts allow the devices to operate and convert the light signal to an electrical current without an external voltage bias. The power consumption can be reduced considerably because no voltage bias is applied, allowing detection anywhere and anytime. Both zero-bias photodetectors and solar cells operate based on photovoltaic effects, but differ in that the two devices target photodetectors and energy conversion, respectively.

This review revisits recent progress in high-sensitivity and high-speed photodetection, focusing on low power, zero-bias operation. Furthermore, various strategies have been introduced, from the realization of zero-bias operation through various structural strategies to strategies using emerging material groups, including organic semiconductors, metal oxide semiconductors, graphene, and TMD, such as MoS_2_ and WSe_2_. In addition, this paper introduces anisotropic structures and polarization sensing applications, which have recently been highlighted, and proposes strategies to implement them through various papers. This review clarifies the advantages of zero-bias-based photodetector devices against several challenges and opportunities.

## 2. Zero-Bias Operation Photodetector Devices

### 2.1. Homo-Material-Based Design for Zero Bias Photodetectors

**Figure 1:**
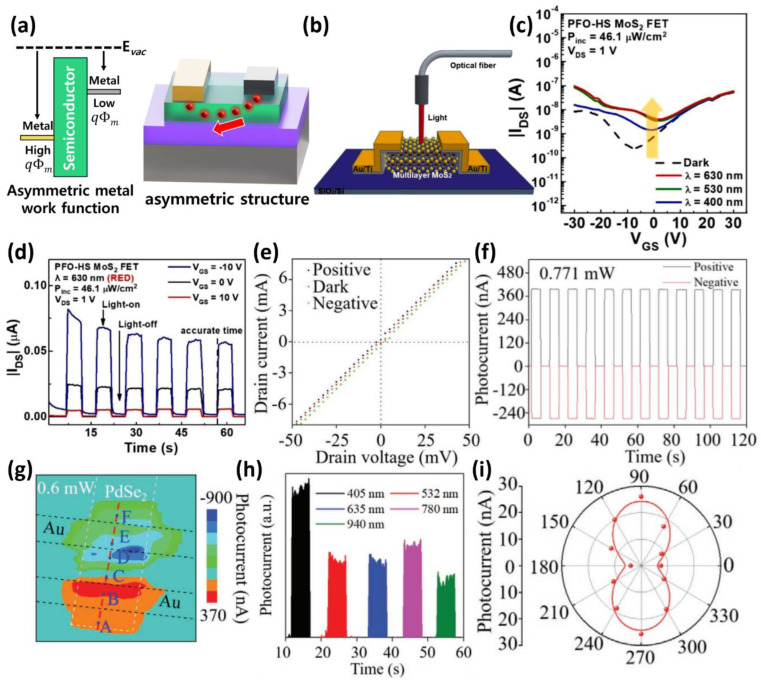
(**a**) Schematic image of zero bias operation using asymmetric work function electrode or asymmetric electrode structure. (**b**) Schematic diagram of PFO-doped MoS_2_ phototransistor and light irradiation measurement. (**c**) Transfer curve change for each red, green, and blue light source of a PFO-doped MoS_2_ phototransistor according to the gate voltage. (**d**) Photoswitching behavior characteristics of PFO-doped MoS_2_ device according to gate voltage variation (V_GS_ = −10, 0, 10 V) in red (630 nm) light irradiation (adapted from [63] with permission from the Elsevier). (**e**) Output characteristics of a device under light irradiation conditions (black and cyan solid lines) and in the dark (solid red line). (**f**) Photocurrent response of laser spot pulses irradiated to the upper and lower interfaces of the PdSe_2_/Au. (**g**) Scanning photocurrent microscopy (SPCM) image mapping of the PdSe_2_ in light of 532 nm, 0.6 mW, and zero bias. (**h**) Pulse response at broadband light wavelengths of PdSe_2_ photodetectors. (**i**) Anisotropic photocurrent response to linearly polarized 532 nm light and the anisotropy ratio is 1.3 (adapted from [64] with permission from the John Wiley and Sons).

The zero bias operating characteristics of homo-material-based photodetectors are generally caused by the asymmetry of the current caused by the asymmetric electrode structure or material (Figure 1a). Transition metal dichalcogenides (TMDs) have been studied widely because of their high and unique photoresponse characteristics, tunability of the properties with thickness, high charge mobility, and lattice characteristics [56,57,58,59]. In particular, the fabrication of MoS_2_- or WS_2_-based photodetectors that require an external power source has been reported. One of the challenges of TMDs is the challenging nature of the large-area manufacturing process for most of the TMD materials [60,61,62].

In 2021, Lee et al., reported a MoS_2_ zero-bias ambipolar photodetector [63]. MoS_2_-based n-type transistors obtained through flake exfoliation-induced ambipolar operation through p-type doping and heat treatment using poly(9,9-di-n-octylfluorenyl-2,7-diyl (PFO) and reported improved photoresponse at a gate bias of 0 V (Figure 1b). Transistors based on MoS_2_ flakes with a top contact structure exhibit typical n-type operation. On the other hand, a high current in the p-type region was achieved through PFO doping. Furthermore, MoS_2_ in the pristine state does not show photoresponses at 400 nm, 530 nm, and 630 nm, while PFO-doped MoS_2_ shows photoreactivity with p-doping (Figure 1c). Because MoS_2_ has a van der Waals interface, it is difficult to combine with PFO chemically. The core-level peak was not changed by analyzing p-doped PFO-MoS_2_ by X-ray photoelectron spectroscopy (XPS). UV-vis absorption analysis was performed on PFO to analyze the low photoreactivity of 400 nm. This reduction in photoreactivity resulted from the absorption of the 400 nm wavelength in the PFO layer under the optimized temperature conditions. As a result, the operation was performed at a gate bias of 0 V in the irradiated pulse of red light at 630 nm, as shown in Figure 1d.

Recently, PdSe_2_ has been studied and is a TMDs material similar to MoS_2_. PdSe_2_ has unique properties, such as electrical anisotropy, owing to its pentagonal puckered two-dimensional structural properties. Li et al., implemented a zero bias photodetector capable of detecting broadband wavelength (405–940 nm) based on PdSe_2_ flakes synthesized through chemical vapor deposition (CVD) [64]. When light is applied to the device, the electron–hole pairs generated inside PdSe_2_ by the photothermoelectric (PTE) effect exhibit a zero-bias photoreaction (Figure 1e). Time-resolved photocurrent spectroscopy performed to analyze photocurrent generation revealed unique properties. As shown in Figure 1f, the photocurrent of the fabricated device is positive when the position of the irradiated laser spot illuminates one end of the device but becomes negative when it is at the other end. These characteristics are more evident by scanning photocurrent microscopy (SPCM). The difference in photocurrent generated by each upper and lower electrode is shown in the mapping image of Figure 1g. As a result, the PdSe_2_ photodetector generates its power and exhibits a photoresponse in various visible and infrared regions, as shown in Figure 1h. In addition, it exhibited an anisotropic photocurrent response owing to the anisotropic structure of the synthesized PdSe_2_ flakes (Figure 1i).

**Figure 2:**
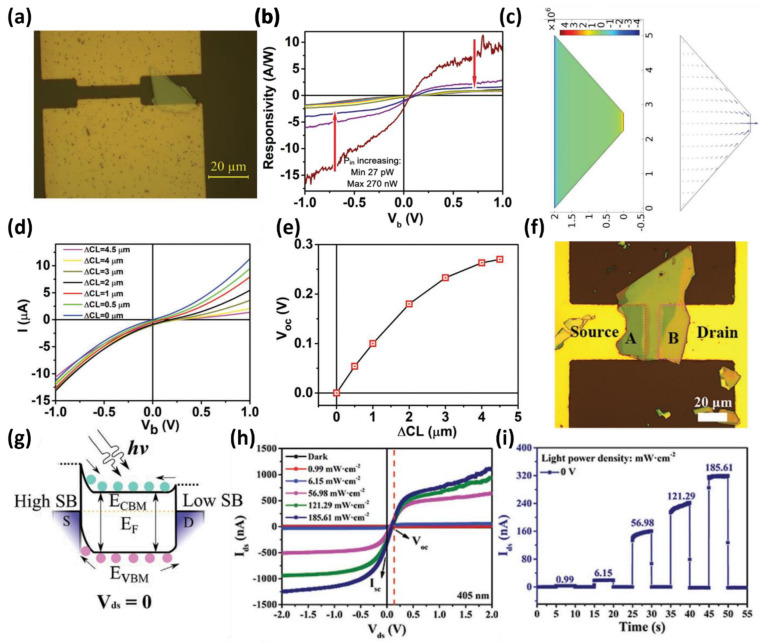
(**a**) Optical microscopy image of the fabricated WSe_2_ flake-based devices with asymmetric electrode contact area. (**b**) Responsivity of the device as a function of voltage bias at different intensities of light irradiation (27 pW to 270 nW). (**c**) Simulated photodetector electric field distribution with 4.5 µm of ΔCL and zero bias. (**d**) I–V curves of various contact length differences for an asymmetric WSe_2_ photodetector. The narrower contact length ranged from 0.5 to 5 µm. (**e**) Dependence of V_OC_ on different ΔCL length variations (adapted from [65] with permission from the John Wiley and Sons). (**f**) Optical microscope image of the designed WS_2_-based photodetector. (**g**) Band diagram of the device showing the asymmetry of the Schottky barrier due to the trap state on the Au surface and the asymmetric thickness of WS_2_ at an external bias of 0 V. (**h**) I–V curves of the WS_2_ photodetector under 405 nm power densities, ranging from 0.99 to 185.61 mW cm^−2^. (**i**) Photoresponse curves at various light power densities at V_DS_ = 0 V (adapted from [67] with permission from the John Wiley and Sons).

Furthermore, previous studies reported that the intrinsic characteristics of a semiconductor were maintained, and a self-powered operation was implemented through variations in the contact area or material of the electrode. Zhou et al., used the asymmetry of WSe_2_ flakes to implement a photodetector in a metal-semiconductor-metal (MSM) structure with a low dark current and showed that the self-powered operation could be controlled through simulation [65]. The photovoltaic effect used as the primary mechanism for WSe_2_-based photodetectors is caused by asymmetric contact lengths similar to reports on silicon using electrodes with different areas [66]. The morphology of the asymmetric WSe_2_ flakes resulted in asymmetric charge injection from both electrodes (Figure 2a). As shown in Figure 2b, the fabricated device exhibited a photoresponse (A/W) of 2.31 A/W and high detectivity of 9.16 × 10^11^ Jones at zero bias. As the contact length difference (ΔCL) increases, the open-circuit voltage (V_OC_) increases, with a concomitant increase in photocurrent at zero bias. The authors simulated I–V curves at various ΔCL to analyze the change in device properties quantitatively according to the change in ΔCL, as shown in Figure 2c,d. As a result, the V_OC_ tended to increase as the change in ΔCL increased (Figure 2e). These structural and electrical tendencies are expected to be applied widely to various semiconductor materials, including TMDs, such as WSe_2_, with systematic geometry engineering.

Gao et al., used the difference in contact area and thickness of the WS_2_ material to obtain a zero-bias-driving optical response [67]. Compared to existing p–n junction-based photodetectors, this method can reduce the number of processes because of the absence of heterogeneous structures. As shown in Figure 2f, the fabricated device has an asymmetric contact area due to the irregular geometry and thickness of the flakes. The asymmetric contact area produces an asymmetric hole trap site between WS_2_ and the metal electrode surface in the WS_2_ transfer process using PVA. The asymmetric trap forms an asymmetric Schottky barrier difference inside the WS_2_, forming an internal potential difference even at zero bias, and moving the carriers generated by the irradiated light (Figure 2g). As a result, the WS_2_-based device exhibits misaligned I_SC_ and V_OC_, as shown in Figure 2h. The misalignment of I_SC_ and V_OC_ increases as the light intensity (405 nm) increases (Figure 2i).

**Figure 3:**
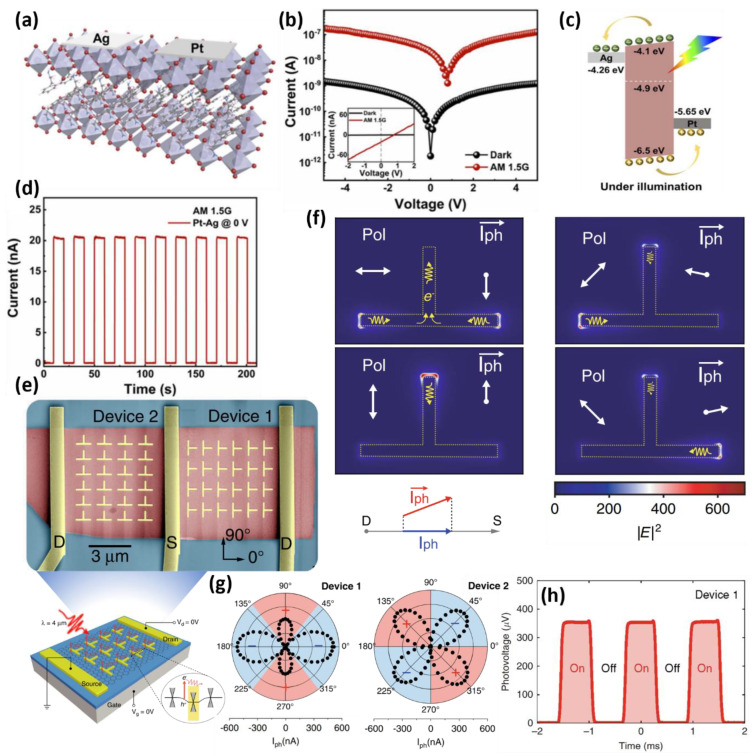
(**a**) Schematic diagram of a photodetector fabricated with 2D perovskite and Pt, Ag asymmetric electrodes. (**b**) the I-V curves of Pt-Ag photodetector in the dark and under a solar simulator. (**c**) Band diagram of the presented device, due to the asymmetric electrode, an internal potential difference is generated. (**d**) Photocurrent response to a solar pulse signal in zero bias operation (adapted from [68] with permission from the Royal Society of Chemistry). (**e**) Schematic diagram and optical microscopy image of the fabricated metamaterial-based graphene photodetector. (**f**) Metamaterial structure that performs a polarization detection for a polarized light input, and (**g**) the resulting detected polarization characteristics. (**h**) Laser pulse-induced photocurrent response of the fabricated device to a 4 µm mid-infrared laser (adapted from [72] with permission from the Springer Nature).

Self-powered operation using the difference in the work function of asymmetric electrode materials has been reported. Yan et al., reported an infrared photodetector with high stability and efficient optoelectronic properties using a 2D layered organic-inorganic hybrid perovskite material (Figure 3a) [68]. Instead of using a conventional p–n junction, in the MSM-based structure, asymmetric electrodes with different work functions form an embedded electric field, and the resulting Schottky junction effectively separated the photogenerated carriers without external bias. As a result, as shown in Figure 3b, the I–V curve of the device deviates from the origin of the coordinates under illumination with a low dark current in the dark state, resulting in an apparent photovoltaic effect. This feature was not observed in devices using identical metal electrodes with symmetrical work functions. The electron–hole pairs formed inside the perovskite material through light can be interpreted by the band diagram (Figure 3c). The different work functions of both electrodes form a different type of Schottky barrier at the interface between the electrode and the semiconductor, forming a built-in potential. Each electron–hole pair generated by light with energy above the bandgap ejects an electron through Ag above the Fermi level of the perovskite, and discharges a hole through Pt below the Fermi level. This charge transport process does not require an external bias because it is caused by a built-in potential difference. Figure 3d shows the photodetector response to the light pulse, and high responsiveness of 114.07 mA/W and high detectivity of 4.56 × 10^12^ Jones were confirmed without an external power supply.

On the other hand, a strategy for improving the photoreactivity using a semiconductor material structure or an upper light absorption structure, as well as improving the intrinsic photoreactivity of the semiconductor material, has been proposed to improve the efficiency of the photodetector [69,70,71]. Wei et al., designed a graphene-based photodetector with high sensitivity and zero bias behavior using the bulk photovoltaic effect (BPVE) [72]. Metamaterial-based nanoantenna with highly engineered optical properties for photodetectors enable the detection of the polarization of irradiated light in a single device, and simulations have been performed on these operations (Figure 3e). Metamaterial-based Au electrodes can improve light absorption considerably using plasmon resonance. The photocurrent generated by the plasmonic effect causes a current to flow in a specific branch through the T-shaped antenna, depending on the polarization characteristics of the irradiated light. These photocurrents generate currents in the vector sum direction, as shown in Figure 3f. The current in the vector sum direction can be considered equal to the current in the corresponding direction between the source and the drain electrode. The degree of polarization of light can be inferred from the direction and magnitude of these currents (Figure 3g). This phenomenon is suitable for 0 V operation because metamaterials and the resulting plasmon effect provides a significant photocurrent. As a result, the device detects the polarization of light in a single device and generates a high photovoltage, as shown in Figure 3h, which successfully exhibits photodetector characteristics. This is the first BPVE mechanism-based photodetector using metamaterials.

**Figure 4:**
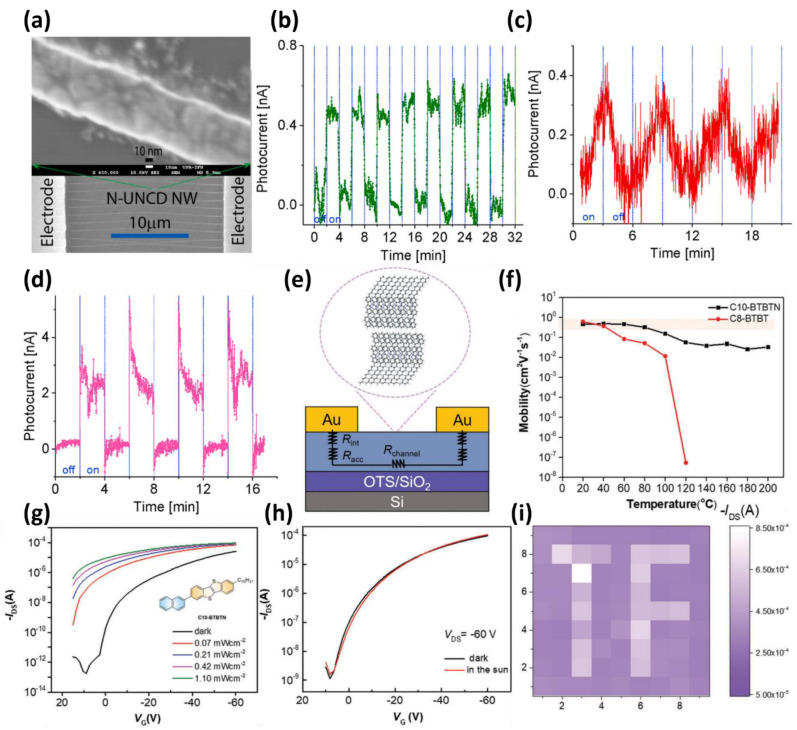
(**a**) SEM image of N-UNCD film and AFM morphology analysis image of the fabricated nanowires. The operation of the proposed N-UNCD NW array photodetector at (**b**) 250 nm, (**c**) 300 nm, and (**d**) 350 nm under the zero-bias condition (adopted from [73] with permission from the Elsevier). (**e**) Schematic diagram of organic phototransistor with C10-BTBTN channel layer with top-contact and bottom-gate structures. (**f**) Comparison of mobility stability of organic semiconductor materials (C8-BTBT and C10-BTBTN) according to temperature. (**g**) Transfer characteristics of C10-BTBTN phototransistors measured with 266 nm laser at various laser intensities. (**h**) Transfer curves of phototransistors compared to under sunlight and dark conditions. (**i**) Current mapping image of 9 × 9 array based on C10-BTBTN phototransistor (adopted from [75] with permission from the Wiley-VCH GmbH).

Furthermore, improvements in the aspect ratio and photoresponse by editing the semiconductor structures have been reported. Pacheco et al., reported a zero-bias UV photodetector using an ultrananocrystalline diamond (UNCD)-based nanowire array [73]. The UNCD transformed from film to nanowire structures through etching enables the formation of high photocurrent because of its high aspect ratio (Figure 4a). Through appropriate nitrogen doping in the synthesis process, the band gap of UNCD can be controlled quantitatively, and UNCD films for photodetectors that are selective for various UV wavelengths can be realized. The author of this paper adjusted the band gap of the UNCD to a target of 350 nm UV light through quantitative nitrogen doping. As a result, under the same light intensity condition of 1 mW/cm^2^, high-energy light with wavelengths of 250 nm and 300 nm showed low photocurrents of 0.26 A/W at 250 nm and 0.32 A/W at 300 nm, respectively, as shown in Figure 4b,c. On the other hand, at the same light intensity of 1 mW/cm^2^, a relatively low energy light of 350 nm wavelength exhibited a high photocurrent of 2.0 A/W due to the selective characteristics of the photodetector (Figure 4d). In addition, at wavelengths over 400 nm, the UNCD film can barely generate a photocurrent because of the lower energy than the bandgap. This characteristic enables the fabrication of UV detectors with a selectivity that can detect only the required wavelength alone through reasonable nitrogen doping concentration control. This UNCD film can be used for photodetector applications with high selectivity in various UV regions through nitrogen doping.

The phototransistor may have higher sensitivity than conventional photodetectors because the transistor amplifies detected light. On the other hand, the power consumption can be increased because an additional gate bias is required. Therefore, phototransistors that can be turned on by light at 0 V or low gate voltage bias are attracting attention for future applications. Li et al., fabricated a solar blind phototransistor (SBPT) using an oxygen annealing process and a recessed gate structure [74]. They reported that SBPT remains OFF in the normal state (non-bias), thanks to this unique annealing and gate structure. Oxygen annealing further depletes electrons in the β-Ga_2_O_3_ channel, increasing the threshold voltage of the device. The recessed gate omits the SiN insulating film, allowing more photons to reach the β-Ga_2_O_3_ channel, providing a large photocurrent to be generated. On the other hand, when the light of the solar blind area (here, ultraviolet light) passes through the gate electrode and the insulating film is made to be thin, the SBPT has a high photo-dark current ratio (PDCR) of 1.5 × 10^6^ at a gate voltage of 0 V because of the intense photoreaction in the β-Ga_2_O_3_ channel. These characteristics were measured at a high-energy light pulse of 254 nm. As a result, with a high external quantum efficiency (EQE) of 6.4 × 10^7^, the photocurrent showed a linear increase with increasing light intensity at a drain voltage of 15 V under a gate bias condition of 0 V or showed an increase in photocurrent as the drain voltage was increased. Furthermore, the fabricated device has a high reactivity of 1.3 × 10^7^ A/W and a detectivity of 4.8 × 10^18^ Jones, showing the highest performance among previously reported β-Ga_2_O_3_-based photodetectors.

A photodetector with high photoresponse efficiency and stability was realized by redesigning the photo-sensing and switching channel material of the phototransistor. In 2022, Dong et al., implemented a phototransistor in the solar-blind region using C10-BTBTN, a naphthyl-substituted [1]Benzothieno [3,2-b][1]-benzothiophene (BTBT) derivative (Figure 4e) [75]. C10-BBTTN detected light in the solar blind region more efficiently because of higher thermal stability, large bandgap (3.3 eV), and improved light absorption than conventional BTBT organic semiconductors. Furthermore, C10-BTBTN showed improved thermal stability over previously reported C8-BTBT with p-type mobility of 2.39 cm^2^/V∙s (Figure 4f). In addition, C10-BTBTN showed significantly improved light absorption in the solar blind region compared to C8-BTBT through UV-vis absorption. With this high light absorption, the photodetector was analyzed under UV light at 266 nm (Figure 4g). The transfer characteristic of the photodetector showed a left shift of V_th_, and a high photocurrent at a gate voltage of 0 V was sufficient to perform zero-bias operation. The presented photodetector does not exhibit any response under sunlight that does not include a solar blind area (Figure 4h). As a result, the C10-BTBTN-based solar-blind photodetector selectively operates only in the non-solar region, with a high detectivity of 7.70 × 10^14^ Jones at a wavelength of 266 nm with the possibility of zero-bias operation. The photodetector array in the form of 9 × 9 pixels successfully produced an image of “TF” strings (Figure 4i), and further experiments confirmed that performance degradation did not appear, even on flexible substrates. Improving the photoresponse using these organic substitutions will provide a new strategy for developing optoelectronic materials.

### 2.2. Heterostructure-Based Design for Zero Bias Photodetectors

**Figure 5:**
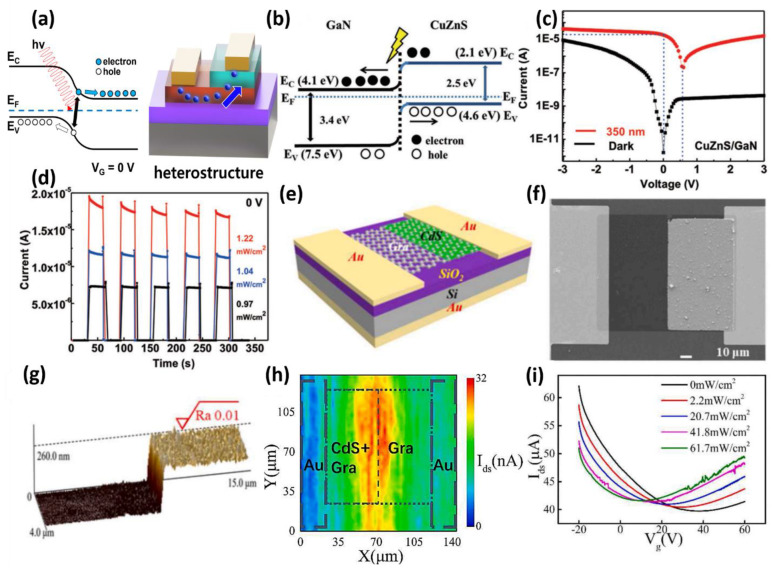
(**a**) Schematic diagram of zero-bias operation using heterostructures. (**b**) p–n junction band diagram of CuZnS/GaN heterojunction device. (**c**) I–V characteristics of CuZnS/GaN heterojunction devices under light (350 nm) and dark conditions. (**d**) Light pulse response with time at various light intensity variations (0.97 mWcm^−2^, 1.04 mWcm^−2^, and 1.22 mWcm^−2^) of the presented heterojunction device (adapted from [82] with permission from Royal Society of Chemistry). (**e**) Structure diagram and (**f**) SEM image of a partial CdS doped graphene photodetector. (**g**) Step AFM image between CdS surface and graphene surface. (**h**) Spatial photocurrent mapping of a photodetector on 532 nm laser irradiation with zero bias at the gate and drain voltages. (**i**) Transfer curve of a photodetector according to gate voltage sweep and light intensity (adapted from [83] with permission from the Elsevier).

Combinations of various materials, including heterojunctions, can form energy band bending in the junction region and allow photoinduced electron–hole pairs to flow easily [76,77,78] (Figure 5a). Therefore, photodetectors based on various heterogeneous structures, including p–n junctions and n–n junctions, have been reported [79,80,81]. Zhang et al., achieved a photodetector with a high photocurrent and rapid response speed through an asymmetric heterojunction using CuZnS and GaN [82]. The CuZnS used in the device has a photoreaction of UV light (350 nm), but GaN does not show photoreaction. On the other hand, a p–n junction device integrating p-type CuZnS and n-type GaN shows zero-voltage behavior with the band diagram, as shown in Figure 5b. The electrons and holes of the electron–hole pair generated by light flow separately from the valence band of CuZnS and conduction band of GaN through the built-in potential of the p–n junction, respectively. As a result, the CuZnS/GaN device showed an I–V curve, as shown in Figure 5c. Compared to the dark state, the I–V curve is shifted to the right by 350 nm light, showing a high current at 0 V bias. The presented device has a high photocurrent of 19 µA, a large on/off ratio of 3 × 10^8^ and improved detectivity of 8 × 10^13^ Jones, as shown in Figure 5d, and provides a textbook-like guideline for zero bias using p–n junctions. Gao et al., realized a graphene photodetector that operated with reasonable responsiveness at zero bias through CdS nanocrystal doping and optimized the properties of the photodetector with respect to the doping area ratio (Figure 5e) [83]. These doping solutions improve the photoresponse of two-dimensional materials and make them high candidates for photodetector materials. As shown in Figure 5f,g, a partially doped CdS nanocrystal layer injects electrons from the electron–hole pairs generated by light into the graphene and raises the Fermi level of graphene. This local adjustment of the Fermi level causes a potential difference inside the same single graphene layer and allows a current at a drain voltage (V_DS_) of 0 V to flow. The current change at the moment of light irradiation was observed through photocurrent mapping to confirm the electron injection from CdS nanocrystal into graphene. Consequently, a high photocurrent was detected in the red region of the graphene/CdS and graphene layer junctions in Figure 5h. These can be attributed to the exchange of electron–hole pairs produced by CdS nanocrystals. In addition, these results are shown through the transfer curve of the photodetector according to the gate voltage in Figure 5i. In the transfer curve, the current difference of the photodetector occurred according to the intensity of light irradiated at the gate voltage of 0 V. The current difference at 0 V_GS_ is sufficient to open the possibility of zero-bias operation. The response results according to the intensity of light indicate that as the intensity of light increases, more photocurrent is generated with a large potential difference inside graphene due to more electron injection. As a result, the device exhibited a response of 0.26 A/W and a quantum efficiency of 51.5% at the maximum optical power density for a modulated specific gate voltage at zero bias.

**Figure 6:**
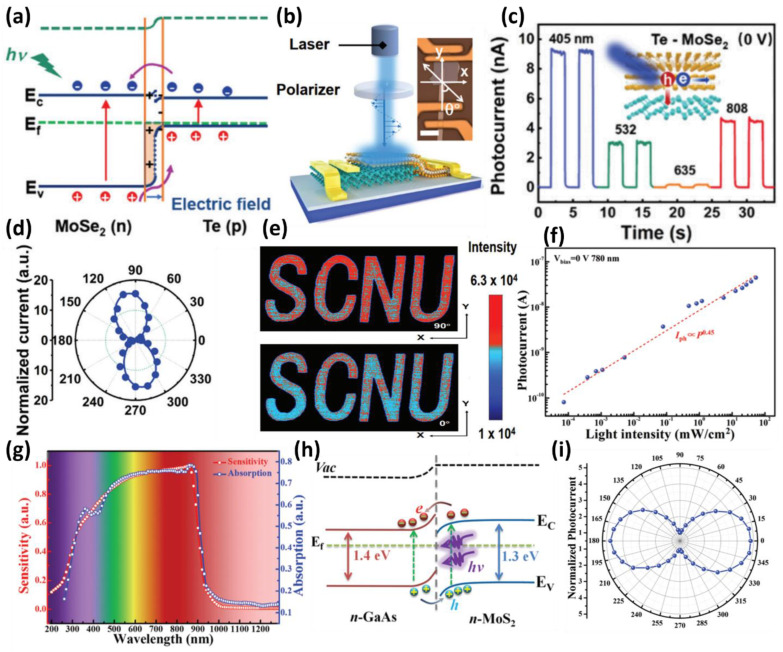
(**a**) Energy band diagram and electron–hole pair transport of Te and MoSe_2_ heterojunction for light irradiation at 0 V. (**b**) Schematic and optical microscopic image of the fabricated Te and MoSe_2_ heterojunction photodetector and schematic diagram of the polarization detection sequence. (**c**) Photoresponse of the photodetector under various light wavelengths at zero-bias voltage. (**d**) Anisotropic photocurrent characteristics shown in the device for irradiated light of 405 nm under zero-bias voltage conditions. (**e**) Images obtained through a polarization imaging measurement system using a heterojunction device presented under conditions polarized at 0° and 90°. (**f**) Plot of the photocurrent as a function of light intensity (adapted from [88] with permission from the Royal Society of Chemistry). (**g**) Response and absorption spectrum according to the wavelength of the device in the heterojunction structure using MoS_2_ and GaAs. (**h**) Energy band diagram and carrier transport of the MoS_2_ and GaAs heterojunction. (**i**) Polarization characteristics of photocurrent in MoS_2_/GaAs heterojunction photodetector (adapted from [89] with permission from the Royal Society of Chemistry).

The detection of polarized light is one of the well-known fields of light sensing due to its potential applications in various fields, such as camera imaging and AI image analysis in the future [84,85,86,87]. Therefore, the realization of a photodetector using a heterostructure can be considered an interesting application. Zhao et al., fabricated a p–n junction diode-based photodetector using n-type MoSe_2_ and p-type Te [88]. The fabricated photodetector has a band diagram of the p–n junction, as shown in Figure 6a. In the heterojunction p–n junction structure, a built-in electric field is generated due to the difference in the Fermi potential of the two materials. The electron–hole pairs produced by light irradiation are moved to both sides by the electric field and form a high photocurrent. The device showed that zero-bias operation in the visible and infrared regions was possible through self-power operation at 0 V, as shown in Figure 6b, with time-dependent pulse operation. The polarization photoresponse characteristics of the fabricated Te/MoS_2_-based device were investigated (Figure 6c). The fabricated device responded strongly to a specific polarized light. This anisotropic photocurrent was derived from the Te layer due to the polarized photodetection and in-plane electrical transport characteristics and exhibited a large anisotropy ratio of 16.39, as shown in Figure 6d. Polarized images were detected by irradiating polarized light at various angles using a photodetector. The output polarization image mapping successfully acquired images as in Figure 6e only at 0° and 90°. Through this, the presented multilayer Te/MoSe_2_ heterojunction photodetector can be considered a potential candidate for a polarization imaging system.

In the same context, Jia et al., fabricated a MoS_2_/GaAs heterojunction-based photodetector by MoS_2_ film transfer. They demonstrated a broadband photoresponse from near-infrared to deep-ultraviolet with zero-bias operation [89]. The fabricated MoS_2_/GaAs heterojunction structure showed a stable response depending on the wavelength and intensity of light at 0 V without an external bias and had a linear response even at a low light intensity of 73 nWcm^2^ (Figure 6f). In addition, the photodetector showed high reactivity of 35.2 mA/W, stable detectivity of 1.96 × 10^13^ Jones, and rapid response with a rising and falling time of 3.4 ms and 15.6 ms at zero-bias conditions. As shown in Figure 6g, the photoreaction started at approximately 870 nm, which corresponds to the bandgap of GaAs, indicating that GaAs occupies a large portion of light absorption and photocurrent generation. These microscopic behaviors were analyzed in detail through the band diagram, and the n–n junction of n-type MoS_2_ and n-type GaAs shows a typical band diagram, as shown in Figure 6h. Electrons and holes generated by the irradiated photons diffuse through the conduction and balance bands of GaAs and MoS_2_, respectively, enabling zero-bias operation. Furthermore, the author investigated the polarization response of a photodetector fabricated by considering the anisotropy of the crystal structure of the 2D MoS_2_ film. As a result, the photocurrent generation was largely due to the polarization angle with a peak-to-valley ratio of 4.8 (Figure 6i). This polarization sensitivity will be considered a suitable candidate for next-generation optical applications.

**Figure 7:**
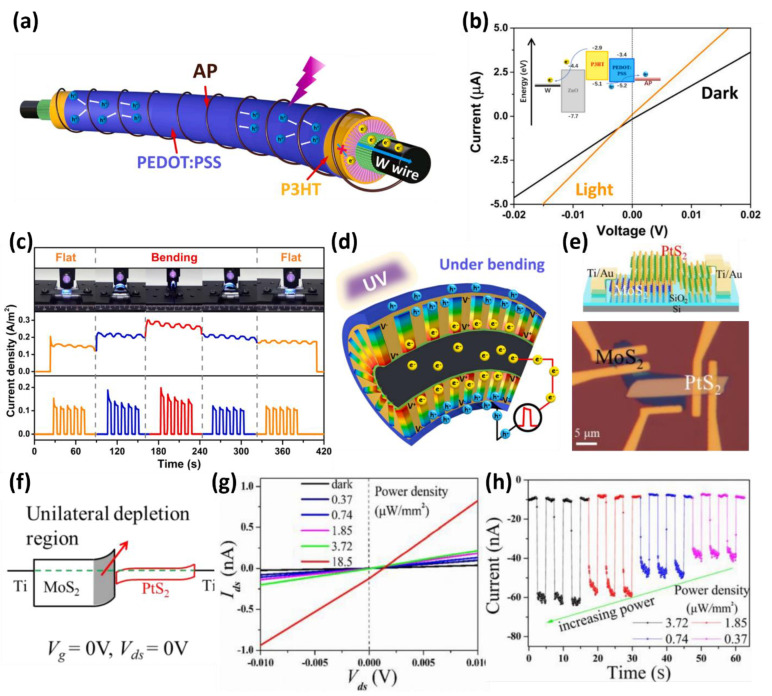
(**a**) Illustration of optically induced carrier transport inside a vertical heterostructure-based fiber-like photodetector. (**b**) I-V curves under 365 nm illumination and dark conditions with energy band diagram of the W/ZnO/P3HT/PEDOT:PSS/AP vertical heterojunction. (**c**) Changes in the photoresponse of the photodetector according to the bending of the wire structure device under the illumination pulse. (**d**) Schematic diagram of the mechanism of a photodetector using the piezoelectric effect under UV illumination to exhibit an enhanced photo-sensing effect (adapted from [90] with permission from the Elsevier). (**e**) Schematic diagram and optical microscopy image of PtS_2_/MoS_2_ heterostructure photodetector. (**f**) Energy band diagram showing the unilateral depletion region of MoS_2_ in the n–n junction structure of MoS_2_ and PtS_2_. (**g**) Shift of I_SC_ and V_OC_ according to the light intensity at a small bias voltage or a bias voltage of 0 V. (**h**) Photocurrent of the PtS_2_/MoS_2_ devices at various power densities at a relatively low operating voltage of V_ds_ = 0.5 V (adapted from [91] with permission from the Royal Society of Chemistry).

Du et al., used a coaxial p–n junction and a piezoelectric optoelectronic mechanism to achieve self-powered operation without an external power source [90]. Vertically stacked n-type ZnO and p-type P3HT were fabricated in the vertical direction from the central W electrode. Together with the polypyrrole-modified alginate fiber (AP) electrode, they were fabricated in a W/ZnO/P3HT/PEDOT:PSS/AP structure (Figure 7a). As shown in Figure 7b, the fabricated device forms a photocurrent by light at an external bias of 0 V. At this time, in the multi-heterojunction film, due to the high HOMO level of P3HT and the relatively low conduction band of ZnO, electrons pass through the W electrode through ZnO and holes pass through the P3HT and PEDOT: PSS layer to the AP electrode. The performance of the photodetector in the bent state of the wire-type device was investigated (Figure 7b). As a result, the bent device generated higher photocurrent when exposed to light than when it was not bent. This unique increase in photocurrent can be addressed through the piezoelectric phenomenon and current generated by the device bent without light. The improved performance due to this bending showed a photoresponse improvement of 81.2% at a strain of 1.96%. The photoresponse improvement in the bent device was investigated by finite element modeling (FEM). Figure 7c presents a schematic diagram of the piezo-potential analyzed by FEM. The physical bending deformation of the device generates a piezo-potential of the ZnO inside the device, further strengthening the internal electric field of the p–n junction, resulting in more effective electron–hole pair separation (Figure 7d). This behavior provides an excellent guideline for light-sensing-based textile wearable applications.

This ideal structural engineering can be realized through a piezoelectric-based photodetector and by controlling the thickness of a heterostructure and forming a unilateral asymmetric depletion region through bandgap engineering. Zhang et al., revealed a photodetector based on van der Waals heterostructures (vdWHs) capable of photodetection at zero bias through an n–n heterojunction structure using PtS_2_ and MoS_2_ [91]. The PtS_2_, a recently discovered TMD material, has received widespread attention owing to its tunable bandgap of 1.6 eV and 0.25 eV in the monolayer and bulk states, respectively. Figure 7e shows an optical microscopy image, along with a schematic illustration of the fabricated device. In this study, the junction of the wide bandgap of MoS_2_ and the thin bandgap of PtS_2_ were designed to have a unilateral depletion region in MoS_2_, as shown in Figure 7f. This was attributed to the narrow bandgap of PtS_2_ in the n–n-type heterojunction, which has a slight heterointerface barrier for photoinduced carriers. Therefore, this type i heterojunction structure has very high carrier mobility. As a result, the PtS_2_/MoS_2_-based photodetector obtained right-shifted open-circuit voltage (V_OC_), and high short-circuit current (I_SC_). These results are considered to have performed a complete self-power operation at a gate bias of 0 V and a drain voltage of 0 V (Figure 7g). In addition, the device obtained an ideal linear photocurrent increase as the light power density increased (Figure 7h) and a rapid response time of 24 ms and a fall time of 21 ms, respectively. These studies are ideal guidelines for developing novel optoelectronic devices using 2D-tmd-based unilateral depletion region and narrow bandgap.

## 3. Conclusions

This review provided an overview of recent advances in high-efficiency photodetectors based on zero bias (self-power) or low-power operation. In particular, in the homo-material-based photodetector, the β-Ga_2_O_3_-based device showed a high photoresponsivity of 1.30 × 10^7^ A/W with a gate bias of 0 V in the UV-C light region. Heterostructure-based photodetector is based on MoS_2_/PtS_2_ and exhibits excellent photoresponsivity of over 403 A/W at 0 V source-drain bias in the visible region. The performance parameters of each of these devices are listed in Table 1, respectively.

Zero-bias operation is a widely requested performance parameter in most applications requiring power consumption issues, including the Internet of Things and low-power image sensing. However, there are still challenges to overcome before this zero-bias drive can be applied to commercial applications.

1. Limitations of homo-material-based photodetectors: Homo-material-based photodetectors can be implemented through simple structural modifications and low process cost because of the small number of processes, but their limited bandgap physically limits their maximum efficiency due to Shockley–Queisser limitations [92,93,94,95]. These challenges can be addressed using heterogeneous semiconductor materials or by proper control and combination of semiconductor materials.
2. Low photoselectivity issue: In general, because photodetectors depend on the bandgap of the semiconductor material, they can cause issues in selectivity for wavelengths with energies above the bandgap. Therefore, the development and standardization of screening layers capable of absorbing unnecessary wavelengths must be accompanied.
3. Insufficient reproducibility of high-performance photodetectors: TMD has been actively studied in high-sensitivity and high-speed photodetectors owing to its strong light response. On the other hand, in general, implementing zero bias by controlling the contact area or thickness of the TMD flakes is strongly dependent on coincidence. For this reason, it is necessary to develop stable TMD film synthesis and developing technology or the field of application of TMD materials that can selectively take only the high photoreactivity of TMD.
4. Lack of high-performance light absorption layer materials: Further research efforts on materials capable of absorbing light of various wavelengths and maximizing photoelectric efficiency is needed. Various types of QDs can be used to absorb various light corresponding to different band gaps of the QDs. In addition, by using a multi-layer light absorption layer, light of various wavelengths can be absorbed in multiple layers to improve efficiency.
5. Uses of quantum dot materials: Using the low bandgap characteristics of InN, PbSe, PbS, etc., it can be used to develop applications of quantum dots or quantum wells that absorb various wavelengths [96,97,98,99,100,101,102,103,104,105,106,107]. However, applications using these quantum dots and zero-bias operation have not yet been systematically investigated. Such efforts could open up more opportunities for the field of optoelectrical devices using quantum dots and quantum wells.
6. Despite these challenges, zero-bias-based photodetector applications could quickly grow-up in sensor areas, such as low-power imaging devices and image analysis applications using AI. For example, most portable camera applications must run with limited battery power. In addition, it eliminates the need for wired connections in outdoor systems, such as CCTV. Detecting polarized light can be effective for image sensing because it can remove scattered sunlight. In summary, zero-bias operation-based photodetectors require continuous development but are valuable materials for future low power and high-efficiency photodetection applications.

## Figures and Tables

**Table 1:** Summary of previously reported zero-bias photodetector applications.

Active Layer	Detection Light	Operating Voltage (V)	Photoresponsivity (A/W)	Detectivity (Jones)	Rise/Decay Time (s)	Ref
Homo-material-based design for zero bias photodetectors						
MoS_2_	Visible	0 V_GS_	-	-	-	[63]
PdSe_2_	Visible	±0.05 V_DS_	1.30 × 10^−3^ A/W	2.55 × 10^7^ Jones	4 µs/14 µs	[64]
Graphene/ Au metamaterial	UV-C to visible	0 V_GS_	1.66 × 10^−2^ A/W	5 × 10^6^ Jones	100 µs/100 µs	[72]
(PEA)_2_PbI_4_ SC	Solar light	0 V_DS_	1.14 × 10^−1^ A/W	4.56 × 10^12^ Jones	1.2 µs/582 µs	[68]
WS_2_	Visible	0 V_DS_	7.77 × 10^−1^ A/W	4.94 × 10^11^ Jones	7 ms/37.2 ms	[67]
Ultrananocrystalline Diamond	UV-A	0 V_DS_	2 A/W	-	1 s/1 s	[73]
WSe^2^	Visible to IR	0 V_DS_	2.31 A/W	9.16 × 10^11^ Jones	-	[65]
C10-BTBTN	UV-C	0 V_GS_	8.40 × 10^3^ A/W	7.70 × 10^14^ Jones	-	[75]
β-Ga_2_O_3_	UV-C	0 V_GS_	1.30 × 10^7^ A/W	4.8 × 10^18^ Jones	-/454 ms	[74]
Heterostructure-based design for zero bias photodetectors						
ZnO/P3HT	UV-A	0 V_DS_	1.56 × 10^−4^ A/W	0.74 × 10^9^ Jones	≈40 ms	[90]
MoS_2_/GaAs	UV-C to IR	0 V_DS_	3.52 × 10^−2^ A/W	1.96 × 10^13^ Jones	3.4 ms/15.6 ms	[89]
Graphene/CdS	Visible to IR	0 V_GS_	0.26 A/W	-	-	[83]
CuZnS/GaN	UV-A	0 V_DS_	0.36 A/W	8 × 10^13^ Jones	0.14 ms/40 ms	[82]
p-Te/n-MoSe_2_	Visible	±0.08 V_GS_	2.11 A/W	2.91 × 10^13^ Jones	22 ms/25 ms	[88]
MoS_2_/PtS_2_	Visible	0 V_DS_	403 A/W	1.07 × 10^11^ Jones	24 ms/21 ms	[91]

## Data Availability

Exclude the statement.
